# Manipulation of a Nuclear Spin by a Magnetic Domain Wall in a Quantum Hall Ferromagnet

**DOI:** 10.1038/srep43553

**Published:** 2017-03-06

**Authors:** M. Korkusinski, P. Hawrylak, H. W. Liu, Y. Hirayama

**Affiliations:** 1 Quantum Theory Group, Security and Disruptive Technologies, National Research Council, Ottawa, K1A 0R6, Canada; 2 Physics Department, University of Ottawa, Ottawa, K1N 6N5, Canada; 3 State Key Lab of Superhard Materials and Institute of Atomic and Molecular Physics, Jilin University, Changchun, 130012, P. R. China; 4 Department of Physics and WPI-AIMR, Tohoku University, Sendai, Japan

## Abstract

The manipulation of a nuclear spin by an electron spin requires the energy to flip the electron spin to be vanishingly small. This can be realized in a many electron system with degenerate ground states of opposite spin polarization in different Landau levels. We present here a microscopic theory of a domain wall between spin unpolarized and spin polarized quantum Hall ferromagnet states at filling factor two with the Zeeman energy comparable to the cyclotron energy. We determine the energies and many-body wave functions of the electronic quantum Hall droplet with up to N = 80 electrons as a function of the total spin, angular momentum, cyclotron and Zeeman energies from the spin singlet ν = 2 phase, through an intermediate polarization state exhibiting a domain wall to the fully spin-polarized phase involving the lowest and the second Landau levels. We demonstrate that the energy needed to flip one electron spin in a domain wall becomes comparable to the energy needed to flip the nuclear spin. The orthogonality of orbital electronic states is overcome by the many-electron character of the domain - the movement of the domain wall relative to the position of the nuclear spin enables the manipulation of the nuclear spin by electrical means.

There is currently a great interest in nuclear spintronics – developing means of storing and manipulating information using nuclear spins in solids^1^,^2^,^3^,^4^,^5^,^6^,^7^,^8^,^9^,^10^,^11^,^12^,^13^,^14^,^15^. A major progress has been achieved recently by experimentally demonstrating electrical detection and manipulation of nuclear spins with spins of electrons in quantum Hall systems^10^,^11^,^12^,^13^,^14^,^15^,^16^,^17^,^18^,^19^,^20^,^21^,^22^,^23^,^24^. However, the microscopic mechanism behind the nuclear spin manipulation with electron spin is not well understood and we fill this gap here.

The major problem in the manipulation of a nuclear spin (black) by the spin of an electron (red) in a given orbital (red) is the difference, ~10^3^, in the energy required to flip the nuclear and electron spins simultaneously, as shown in Fig. 1(a). If the electron spin flips simultaneously with the change of the electron orbital, from blue to red as shown in Fig. 1(a), the difference in Zeeman energies, ΔΕ_z_, can be compensated by the difference in orbital energies. However, the two electronic orbitals, red and blue, need to be orthogonal and zeros in one of the orbital wavefunction make the amplitude of the hyperfine interaction vanish for some positions of the nuclei. If the transition between different orbitals (red and blue in Fig. 1(a)) represents schematically a transition between the degenerate many-body electronic states, for example, spin polarized (spin down) and unpolarized (spin up) domains in the two-dimensional electron gas (2DEG)^10^,^14^,^15^,^16^,^19^,^20^,^21^,^22^,^23^,^24^,^25^,^26^,^27^,^28^,^29^, the initial and final states are spatially separated by a domain wall and cannot both overlap with the nuclear spin. Hence the microscopic mechanism of the hyperfine coupling depends on the many-electron character of the domain wall separating the two electronic phases.

## Model

**Figure 1:**
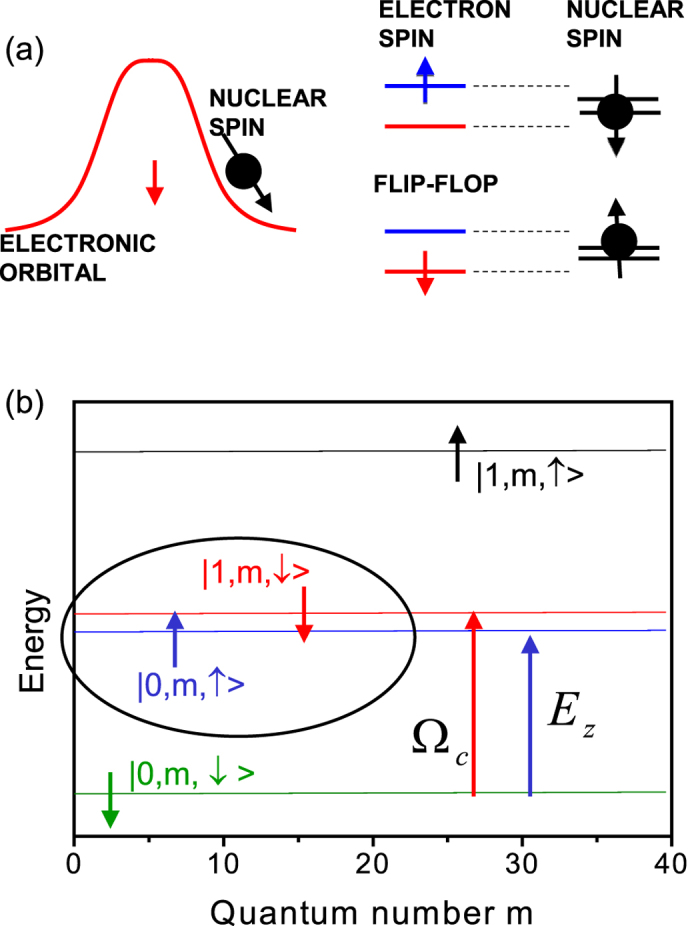
(**a**) Left: Schematic view of the electron and nuclear spin interaction. The red curve represents the charge density of a single spin-down electron orbital, while the nuclear spin is marked in black. Right: The simultaneous flipping of nuclear spin and electron spin involving the electron orbital transition, from blue to red, spin, to match the nuclear and electron spin Zeeman energies. (**b**) The red and blue single-particle electronic states realized in a two-dimensional quantum dot with weak parabolic confinement in a large perpendicular magnetic field, with the cyclotron energy Ω_*c*_ comparable to the Zeeman splitting *E*_*z*_ due to the large electronic Lande factor.

To understand how these contradictions can be overcome we focus on a simple yet general model of a domain wall in a quantum Hall ferromagnet (QHF)^25^,^26^,^27^,^28^,^29^. For the simplest QHF at filling factor ν = 2, recently realized in InSb quantum wells^19^,^20^,^21^, the comparable cyclotron and Zeeman energies result in the degeneracy of spin up electron states of the lowest *n* = 0 Landau level (blue) and spin down electron states of the second *n* = 1 Landau level (red) as illustrated in Fig. 1(b). For the electron to occupy red and blue levels a finite number of electrons filling the lower energy green and blue states, filling factor ν = 2, is needed. Hence the many-body character of the interaction of electronic and nuclear spins of the domain wall in a QHF which we treat exactly^25^,^31^,^32^,^33^, beyond the variational mean field description of the domain wall^26^,^27^,^28^,^29^,^30^. We hence model the ν = 2 state by *N*_*e*_ electrons confined to a finite size quantum Hall droplet (QHD) in a perpendicular magnetic field *B*^31^,^32^,^33^. The electrons interact via the contact hyperfine interaction with a nuclear (impurity) spin 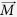 at a position 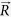. The single electron states are 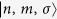 with energies *ε*(*nmσ*), where *n* is the Landau level (LL) index, *m* the intra-LL quantum number, σ = ±1 the electron spin, and the electron Zeeman energy is comparable to the cyclotron energy, 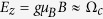 (see Supplementary Material for details). We note that the orbitals 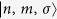 form rings, whose radii increase as 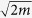 within each LL. With 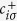 (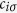) the electron creation (annihilation) operators on the orbital *i* ≡ (*n, m*) and 
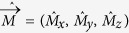
 the spin operator of the nuclear spin, the Hamiltonian of electrons and a localized nuclear spin M can now be written as^31^,^32^,^33^:


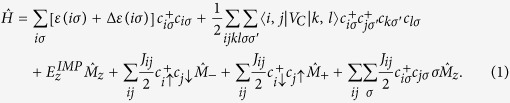


The first term is the electron energy, the second term describes the electron-electron Coulomb interactions and the third term is the Zeeman energy 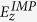 of nuclear spin. The last three terms describe the hyperfine interaction of the electron and the nuclear spin, with the matrix elements 
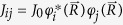
31. Finally, the term 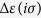 accounts both for the interactions with the positive background and removal of the finite-size effects. This correction is chosen by ensuring that the Coulomb exchange energy is uniform across the QHD and by balancing the total negative charge of the system by an equivalent number of positive charges (see Supplementary Material for details). For clarity, we restrict here the single-particle spectrum to two lowest Landau levels, shown in Fig. 1(b). The lowest Landau level (LLL) orbitals *ε*(*n* = 0, *m*) are drawn in green and blue, while the second Landau level (2LL) orbitals *ε*(*n* = 1, *m*) are drawn in red and black. With the quasi-degeneracy of the LLL spin up orbitals *ε*(*n* = 0, *m*, ↑) (blue in Fig. 1(b)) and the 2LL spin down orbitals *ε*(*n* = 1, *m*, ↓) (red), the energy to flip the spin and change LL orbitals of one electron is comparable with the energy to flip the nuclear spin. However, we have not one but *N*_*e*_ electrons, with the spin-down LLL completely filled, and the quasi-degenerate orbitals of spin-up LLL and spin-down 2LL populated partially.

## Construction of spin domain states

**Figure 2:**
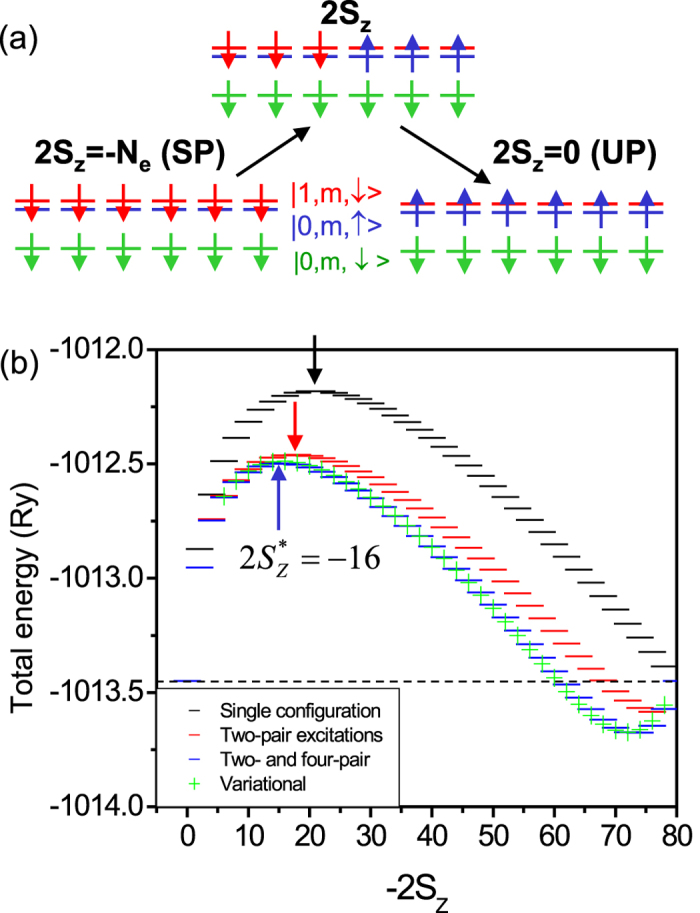
(**a**) The spin-polarized configuration involving two Landau levels (left), the spin-unpolarized ν = 2 configuration (right) and the spin-domain configuration (top). (**b**) The energy of the *N*_*e*_ = 80 electron quantum Hall droplet as a function of the total spin projection S_z_. The spin-unpolarized ν = 2 and fully spin-polarized configurations (black dashed line) are degenerate. The black, red and blue lines denote respectively the energies of the HF configuration and states containing two-pair and two- and four-pair configurations while the green symbols shows results of variational calculation. The arrows mark the highest energy barrier state.

We start by constructing two states, shown in Fig. 2(a). The SP state on the left, 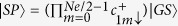 is completely spin polarized, while the UP state 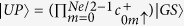 on the right-hand side of Fig. 2(a) is the spin-unpolarized configuration, a finite-size ν = 2 QHD, where 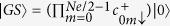 is the spin down polarized reference QHD. The two states have different total spin projections: 2*S*_*z*_ = −80 for 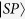, 2*S*_*z*_ = 0 for 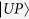, and total angular momenta 
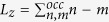
. Flipping the spins in state 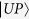 and transferring them to the 2LL generates states with intermediate total *S*_*z*_ and *L*_*z*_. These configurations represent domains of spin-down electrons in the center and spin-up electrons at the edge of the QHD, with a clear domain wall separating them, as depicted in the top panel of Fig. 2(a). We vary 2*S*_*z*_ from 0 to −80 and for each domain wall configuration *S*_*z*_, *L*_*z*_, states 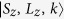 are expanded in two-, four-, and more electron-hole pair excitations:


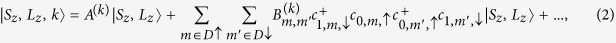


and the electronic part of the Hamiltonian (1) is diagonalized in this basis. Here, 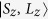 denotes the HF spin-domain configuration. As evident from the second term of Eq. (2), the two-electron-hole pair excitations are formed by flipping the spin of one electron in the spin-down domain without changing its orbital quantum number *m*, while flipping the spin of another electron from the spin-up domain in the same manner.

The number of such low energy excitations quickly grows with size of the system. For example, for *N*_*e*_ = 80 electrons, of which 40 are the spin-polarized LLL background, 20 are in the spin up domain *D*↑, and 20 in the spin down domain *D*↓, there is one fundamental domain configuration, 20^2^ = 400 two-pair excitations, and 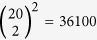 four-pair excitations. The exact wavefuntions 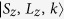 (Eq. 2) in the finite electron-hole number pair approximation may be contrasted with variational, spin and angular momentum non-conserving wavefunction^25^,^26^,^27^,^28^,^29^ 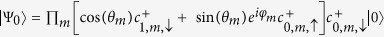 parametrized by the pseudospin 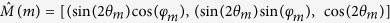 which varies slowly on the magnetic length scale^27^.

## Energy spectra of spin domain states

In the following, we present results of model calculations for the QHD with N_e_ = 80, confining energy 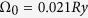, cyclotron energy 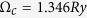, 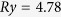 meV, the effective Bohr radius *a*_*B*_ = 12.15 nm, and the characteristic length *l*_*h*_ = 1.219*a*_*B*_.

**Figure 3:**
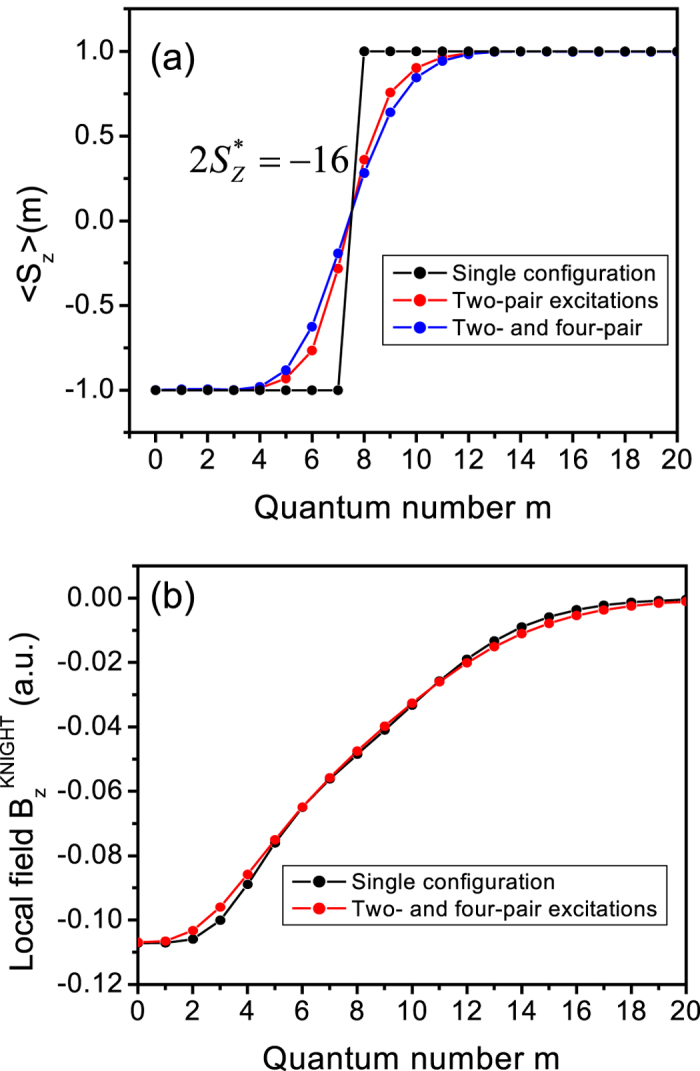
(**a**) The local spin polarization 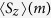 in the state with 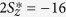 as a function of the orbital quantum number *m* and as a function of the number of pair excitations admixed into the state: zero (black), two pair (red), and two and four pairs (blue). (**b**) The effective Knight field 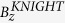 experienced by the nuclear spin as a function of the position of that spin within the quantum Hall droplet. The spin is positioned in the maximum of the lowest-Landau level orbital with quantum number *m*. Black line corresponds to the HF-configuration spin domain state 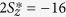, while the result denoted by the red line accounts for the two- and four-pair excitations.

Figure 2(b) shows the energies of the domain-wall configurations as a function of the total spin projection *S*_*z*_, from total 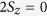 ν = 2 configuration 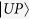 to total 2*S*_*z*_ = −80, fully spin-polarized, configuration 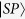, with the Zeeman energy yielding degeneracy of the spin polarized and unpolarized states. The energies of single HF spin-domain configurations (black lines), HF+ two-pair (red lines), and HF+two+four-pair excitations (blue lines) are shown. Increasing spin polarization increases the spin-polarized domain in the center at the expense of the spin-unpolarized domain at the edge of the QHD. The energy of the two domains increases with spin polarization −2*S*_*z*_, reaches its maximum at 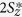 marked in Fig. 2(b) by black arrows, and then decreases. The critical value 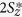 depends on the amount of correlations: it shifts from 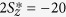 for HF to 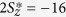 with two- and four-pair excitations included. The variational ground state energies as a function of 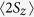, shown in green in Fig. 2(b), compare very well with energies obtained in exact diagonalization with four-pair excitations included. The energy of the 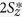 state is the energy barrier needed to flip 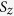 spins. The domain wall character of the 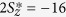 state is illustrated by the spatial dependence of the expectation value of the electron spin, 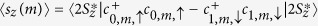, on orbital *m*. Figure 3(a) shows how electron spin projection rotates from down in spin polarized phase to up in unpolarized phase. In the HF approximation (black) we see an abrupt change of spin orientation, inclusion of electron-hole pair excitations leads to a finite width of the domain wall centered on the (*n, m*) = (1, 7) and (*n, m*) = (0, 8) orbitals. The domain wall leads to effective Knight magnetic field 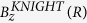 seen by nuclear spins: 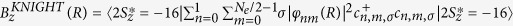, shown in Fig. 3(b) in different levels of approximation. The effective Knight field is large in a spin polarized domain in the center of the QHD, and decreases to zero towards the spin unpolarized domain. Interestingly, we find that the domain wall in the Knight shift is much broader than what might be expected from the electron spin alone, shown in Fig. 3(a).

## Spin domain states interacting with a nuclear spin

**Figure 4:**
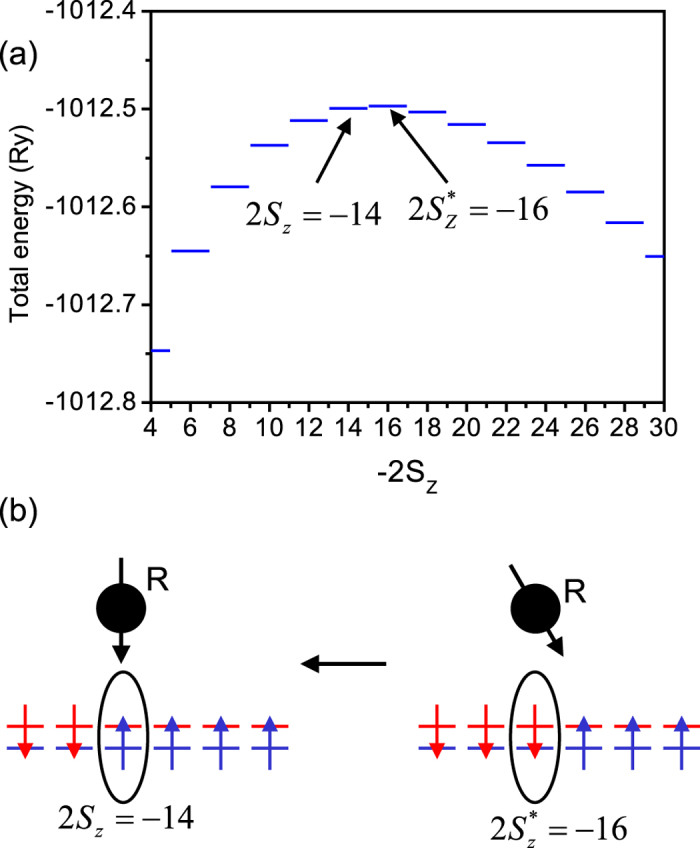
The energies of the electronic quantum Hall droplet as a function of the total spin projection close to the critical value of 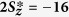. Arrows indicate the initial and final state involved in the flip-flop transition between the electrons and the nuclear spin, discussed in the text, and visualized schematically in panel (**b**).

Let us now discuss the electronic spin flip. We are interested in the energy to flip *one* electron spin in the domain wall state 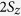, i.e., the difference of energies corresponding to 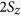 and *S*_*z*_ + 2 configurations. This energy difference is smallest close to the critical value of 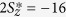 in this illustration, close to the top of the energy barrier. Hence the degeneracy of the domain wall states at the top of the energy barrier, not the degeneracy of the two electronic domains, gives the electron spin flip energy commensurate with the energy needed to flip the nuclear spin, thus enabling the flip-flop process between the electron and the nuclear spins. In Fig. 4 we switch from the initial state 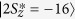, depicted schematically in the right-hand diagram of Fig. 4(b), to the final state 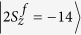, corresponding to one electron spin flip Fig. 4(a). The final state, depicted schematically in the left-hand diagram of Fig. 4(b), is also a domain-wall state, but with the domain wall shifted by one orbital towards the center of the QHD. In this transition, the energy of the electronic system decreases, as shown in Fig. 4(a). As a result, the energy of nuclear spin, residing at position *R*, is increasing with its spin rotating up, as depicted schematically in Fig. 4(b). The probability of this flip-flop process is given by the matrix element of the electron-nuclear interaction part of the Hamiltonian (1):


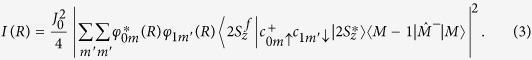


**Figure 5:**
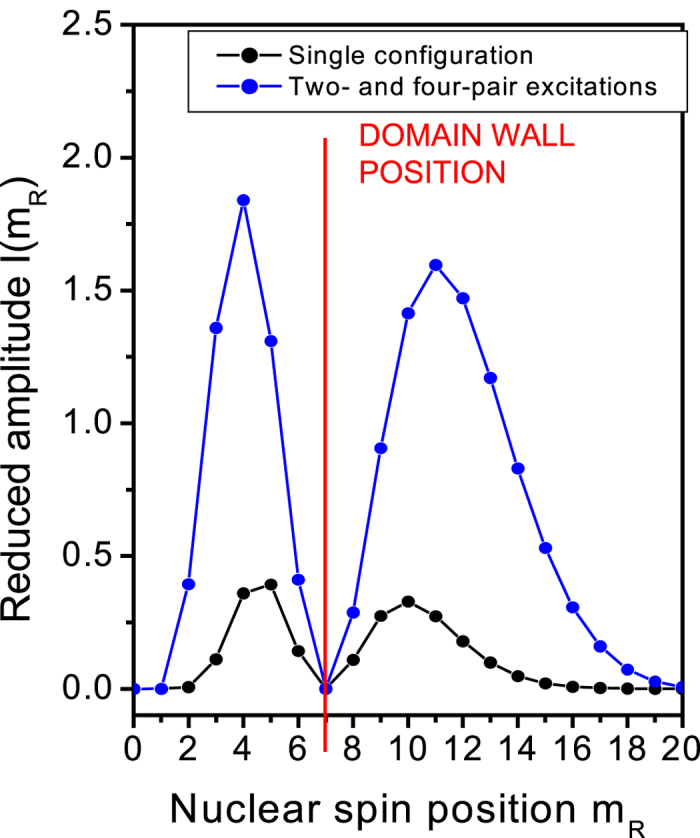
The amplitude of the flip-flop process as a function of the position of the nuclear spin within the quantum Hall droplet for the HF-configuration (black) and correlated electronic state (blue). The nuclear spin is placed at the maximum of the lowest Landau level orbital with the quantum number *m*_*R*_. The red line denotes the position of the domain wall.

Figure 5 shows the reduced amplitude 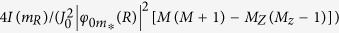 as a function of the position 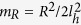 of the nuclear spin. If the domain wall is restricted to HF configurations shown in Fig. 4(b), the only spin flip which converts the 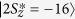 configuration to the 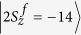 configuration can take place at the domain wall boundary *m *= m^*^ with amplitude given by 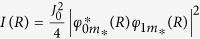. We note that this amplitude is exactly zero at the orbital corresponding to the center of the domain wall (*m*_*R*_ = m^*^ = 7). This results from the orthogonality of the single-particle orbitals corresponding to the initial occupied and final empty electronic state. As the nuclear spin moves away from the domain wall the tail of the wavefunction leads to finite transition probability. Figure 5 shows the amplitude of the electron-nuclear spin flip-flop as a function of position *R* of the nuclear spin. The black line gives the amplitude calculated for the HF single spin-domain configurations only. As we add the correlations (blue line), we see that the amplitude is also zero when the nuclear spin is placed at the center of the domain wall, but the amplitude is significantly enhanced for all other positions of the nuclear spin due to electronic correlations, i.e., transitions within the width of the domain wall are contributing.

## Summary

We presented here a microscopic theory of hyperfine coupling of a nuclear spin with the spins of electrons in a domain wall of a quantum Hall ferromagnet. We showed that the energy of the electronic spin transition in the domain wall can be brought down to the energy needed to flip the nuclear spin while the amplitude, related to the movement of the domain wall, is enhanced by electronic correlations. This understanding opens the way towards predictive theories of nuclear spin manipulation with electron spin, accounting for material parameters, improved treatment of electron-electron interactions, spin–orbit coupling and strong coupling between many nuclear and electron spins^30^,^31^.

## Additional Information

**How to cite this article**: Korkusinski, M. *et al*. Manipulation of a Nuclear Spin by a Magnetic Domain Wall in a Quantum Hall Ferromagnet. *Sci. Rep.* **7**, 43553; doi: 10.1038/srep43553 (2017).

**Publisher's note:** Springer Nature remains neutral with regard to jurisdictional claims in published maps and institutional affiliations.

## Supplementary Material

Supplementary Material
