# Supplementary material for: Manipulation of a Nuclear Spin by a Magnetic Domain Wall in a Quantum Hall Ferromagnet
Source: Sci Rep. 2017 Mar 6;7:43553. doi: 10.1038/srep43553 (PMC5337906; doi:10.1038/srep43553)
Supplement: Supplementary Material [file srep43553-s1.doc]

**Supplementary Material - Manipulation of a Nuclear Spin by a Magnetic Domain Wall in a Quantum Hall Ferromagnet**

M. Korkusinski^1*^, P. Hawrylak^2^, H. W. Liu^3^ and Y. Hirayama^4^

*^1^Quantum Theory Group, Security and Disruptive Technologies, National Research Council, Ottawa, K1A 0R6, Canada;*

*^2^Physics Department, University of Ottawa, Ottawa, K1N 6N5, Canada;*

*^3^ State Key Lab of Superhard Materials and Institute of Atomic and Molecular Physics, Jilin University, Changchun 130012, P. R. China*

*^4^Department of Physics and WPI-AIMR, Tohoku University, Sendai, Japan.*

We study $N_{e}$ electrons confined to a two dimensional quantum dot in a perpendicular magnetic field $B$ interacting with a localized spin $\vec{M}$ at a distance $\vec{R}$ from the center of the dot. In this system, the single particle states $|n,m,\sigma\rangle$ are those of a two-dimensional harmonic oscillator with spin, and have energies $\varepsilon\left(nm\sigma\right)=\Omega^{+}\left(n+\frac{1}{2}\right)+\Omega^{-}\left(m+\frac{1}{2}\right)+\frac{1}{2}g^{*}\mu_{B}B\sigma$, with *n* being the Landau level (LL) index, *m* being the intra-LL quantum number, $\Omega^{\pm}=\sqrt{\Omega_{0}^{2}+\Omega_{c}^{2}/4}\pm\Omega_{c}/2$, $\Omega_{0}$ is the confinement energy, $\Omega_{c}$ is the cyclotron energy, $g^{*}$ is the effective Lande factor, $\mu_{B}$ is the Bohr magneton, and $\sigma=\pm1$ is the electron spin. When the cyclotron energy is much bigger than the confinement energy, the energy spectrum resembles 2D Landau levels, with $\Omega^{+}\approx\Omega_{c}$ and $\Omega^{-}\approx0$. Moreover, the key property of the InSb material is that the electron Zeeman energy is comparable to the cyclotron energy, $E_{z}=g\mu_{B}B\approx\Omega_{c}$.

The Hamiltonian of electrons and a localized nuclear or impurity spin M can now be written as:

(A)

Here, $c_{i\sigma}^{+}$ ($c_{i\sigma}$) is the electron creation (annihilation) operator creating an electron with spin $\sigma$ on the orbital $i\equiv\left(n,m\right)$, while is the spin operator of the nuclear spin. The electronic Coulomb interactions are accounted for in the second term, with the Coulomb matrix elements available in a closed form.

The energy spectrum of the nuclear spin is scaled by the Zeeman energy $E_{z}^{IMP}$. The three last terms of the Hamiltonian describe the interaction between the electron and the nuclear spin. Two of these terms account for the flip-flop process, whereby the electron is scattered from the single-particle orbital *i* to *j* with a spin flip, while the third term describes an analogous scattering process, only without the spin flip. This last term is the origin of the Knight shift of the nuclear spin spectrum due to the effective magnetic field produced by the electron spins.

As we assume the spin-spin interaction to be of the contact form,$J\left(\vec{R}\right)()=J_{0}\delta\left(\vec{R}\right)()$, with $J_{0}$ being the strength of the hyperfine coupling (a material parameter), the spin-spin interaction terms are scaled by the elements $J_{ij}=J_{0}\phi_{i}^{*}\left(\vec{R}\right)()\phi_{j}\left(\vec{R}\right)()$, where the single-particle electron orbitals , and $\vec{R}$ is the position of the nuclear spin.

Finally, the correction term $\Delta\varepsilon\left(i\sigma\right)$ present in the single-particle electron term accounts for the interactions with the positive background, assuring the charge neutrality of the system, and removes the finite-size effects in the manner discussed below.

We restrict the single-particle spectrum to two lowest Landau levels. The lowest Landau level (LLL) orbitals have energies$\varepsilon\left(n=0,m\right)$, while the second Landau level (2LL) orbitals have energies $\varepsilon\left(n=1,m\right)$. The electronic Zeeman energy is comparable to the cyclotron energy, resulting in a quasi-degeneracy of the energies of LLL orbitals $\varepsilon\left(n=0,m,\uparrow\right)$ and the 2LL orbitals $\varepsilon\left(n=1,m,\downarrow\right)$. The energy gap between the Landau levels is therefore comparable with the Zeeman energy $E_{z}^{IMP}$ of the nuclear spin, enabling the flip-flop transitions between the electronic and nuclear spins. For this to take place, however, we have to populate the electronic levels so that the spin-down LLL is filled completely, and the quasi-degenerate orbitals of spin-up LLL and spin-down 2LL are populated partially. We start by constructing two states with $N_{e}$ electrons. The completely spin polarized state, with spin projection $2S_{z}=-N_{e}$, is created by populating the spin-down orbitals of both LLL and 2LL and therefore it can be written as $|SP\rangle=\left(\prod_{m=0}^{Ne/2-1} c_{1m\downarrow}^{+}\right)\left(\prod_{m=0}^{Ne/2-1} c_{0m\downarrow}^{+}\right)|0\rangle$, where $|0\rangle$ denotes the vacuum. The spin-unpolarized configuration is created using the spin-up and down orbitals of the LLL, i.e., $|UP\rangle=\left(\prod_{m=0}^{Ne/2-1} c_{0m\uparrow}^{+}\right)\left(\prod_{m=0}^{Ne/2-1} c_{0m\downarrow}^{+}\right)|0\rangle$. This is a finite-size ν=2 droplet. In order to construct these states with a finite number of electrons, we have to restrict the number of orbitals enumerated by the quantum number *m* in the otherwise infinite Landau levels. As a consequence, there appears an edge of the droplet. This has particularly important consequences in the self-energies of the electrons close to the edge, as they are now surrounded by fewer electrons, as opposed to the electrons close to the center of the droplet. Additionally, the electrons interact with each other and with positive background. We include both these effects by choosing the appropriate corrections $\Delta\varepsilon\left(n,m\right)$ to the single-particle energies, as indicated in the Hamiltonian, Eq. (A). The positive background, compensating for the repulsive electron-electron interactions, is included by $N_{e}$ holes placed in the ν=2 configuration. Furthermore, we balance the exchange self-energy variation across the droplet by introducing a nonuniform potential, which brings the self-energy of the electron on the orbital *(n,m)* down to the value experienced by the central electron *(n,m=0)* at ν=2. We have:

$$\Delta\varepsilon\left(n,m\right)=\Delta\varepsilon_{D}\left(n,m\right)+\Delta\varepsilon_{X}\left(n,m\right),$$

$$\Delta\varepsilon_{D}\left(n,m\right)=-2\sum_{m'=0}^{Ne/2-1} \langle nm,0m'|V_{C}|0m',nm\rangle,$$

$\Delta\varepsilon_{X}\left(n,m\right)=\sum_{m'=0}^{Ne/2-1} \langle nm,0m'|V_{C}|nm,0m'\rangle-\sum_{m=0}^{Ne/2-1} \langle n0,0m'|V_{C}|n0,0m'\rangle$.

Since these corrections are taken with respect to the spin-singlet ν=2 configurations, they are spin-independent. Moreover, the compensation will be complete only for that ν=2 configuration; in any other state (including the fully spin-polarized $|SP\rangle$) the edge effects will weakly reappear.

We consider the configurations with fully occupied spin-down LLL, forming a spin-polarized background. The remaining $N_{e}/2$ electrons are all spin down for the state $|SP\rangle$ and all spin up for the state $|UP\rangle$. The two states have different total spin projections: $2S_{z}=-80$ for $|SP\rangle$, and $2S_{z}=0$ for $|UP\rangle$, counting the spectator LLL spin-down electrons. Also, they are characterized by unique total angular momenta defined as $L_{z}=\sum_{n,m}^{occ} n-m$, where the summation is carried out over the occupied orbitals. By starting with the state $|UP\rangle$ and gradually flipping the spins and transferring them to the 2LL, we can now generate states with intermediate total $S_{z}$ and total $L_{z}$ such that they exhibit a domain of the spin-down electrons in the center of the droplet, and spin-up electrons towards its edge, with a clear domain wall separating them. Unlike the states $|UP\rangle$ and $|SP\rangle$, these domain configurations are not eigenstates of the Hamiltonian, Eq. (A). Indeed, we can flip the spin of one electron from the spin-up domain, thereby transferring it from the orbital $\left(0,m,\uparrow\right)$ to the orbital $\left(1,m,\downarrow\right)$, and leaving behind a hole in the spin-up domain. At the same time we flip the spin of one electron in the spin-down domain, thereby transferring it from the orbital $\left(1,m',\downarrow\right)$ to the orbital $\left(0,m',\uparrow\right)$, and also leaving behind a hole in the spin-down domain. Such a two electron-hole pair excitation will have the same total angular momentum $L_{z}$ and the same total spin projection $S_{z}$ as the original domain configuration, and therefore the two configurations will be coupled by the exchange interaction. In this way we can construct two-, four-, and more electron-hole pair excitations and diagonalize the electronic part of the Hamiltonian (A) in the basis of these configurations. As a result, the ground state of the electron system can be written as:

$|S_{z},L_{z},k\rangle=A^{(k)}|S_{z},L_{z}\rangle+\sum_{m\in D\uparrow} \sum_{m'\in D\downarrow} B_{m,m'}^{(k)}c_{1,m,\downarrow}^{+}c_{0,m,\uparrow}c_{0,m',\uparrow}^{+}c_{1,m',\downarrow}|S_{z},L_{z}\rangle+...$

Here, $|S_{z},L_{z}\rangle$ denotes the fundamental, HF, spin-domain configuration, and the excitations can be constructed for any pair of orbitals, one in the spin-up domain $D\uparrow$, and the other one in the spin down domain $D\downarrow$.

We note that the electronic spin flip can be accompanied by a movement of the electron along the Landau level, as long as the change in angular momentum is compensated by the contrary movement of the electron from the other spin domain. However, such configurations are higher in energy than those described above, because the electrons are then placed on top of the other electrons in the system, and begin to interact with them by direct Coulomb terms, not compensated by the positive background. This is why we do not include such multiply-occupied configurations in our Hilbert space. For example, in a system with $N_{e}=80$ electrons, of which 40 are the spin-polarized LLL background, 20 are in the domain $D\uparrow$, and 20 in the domain $D\downarrow$, there is one HF domain configuration, $20^{2}=400$ two-electron-hole-pair excitations, and $\left(\begin{matrix} 20 \\ 2 \end{matrix}\right)^{2}=36100$ four-pair excitations.

In the following, we present the results of model calculations for the system with $\Omega_{0}=0.021Ry$ and $\Omega_{c}=1.346Ry$. The energy is measured in units of effective Rydberg, which we choose to be $1Ry=4.778$ meV. The distances are measured in the units of the effective Bohr radius, $a_{B}=12.1515$ nm, and the characteristic oscillator length .

**Domain wall.**

Figure 2(b) of the main text shows the energies of the domain-wall configurations as a function of the total spin projection $S_{z}$ from $2S_{z}=0$ (the ν=2 configuration $|UP\rangle$) to $2S_{z}=-80$ (the fully spin-polarized configuration $|SP\rangle$). The Hilbert spaces of the $2S_{z}=0$ and $2S_{z}=-80$ cases consist of only one configuration, while the basis sizes for intermediate spins grow factorially with the sizes of spin domains. The energies depicted in Fig. 2(b) account for the choice of the Zeeman energy, in which the states $|UP\rangle$ and $|SP\rangle$ become degenerate. We have also increased the influence of correlations, computing the energies as single spin-domain configurations only (black lines), adding two-pair (red lines), and two- and four-pair excitations (blue lines). We see that, for each value of $2S_{z}$, the inclusion of correlation effects lowers the energies of the system, but this energy shift saturates quickly with the increase of the number of pair excitations. As the spin polarization increases (the spin-polarized domain in the center increases at the expense of the spin-unpolarized domain towards the edge of the droplet, or alternatively – the domain wall is shifted from the center towards the edge of the droplet), the lowest energy of the system initially increases, reaches its maximum (denoted in Fig. 2(b) by black arrows), and then decreases. The critical value $2S_{z}^{*}$, for which we find the maximum energy, depends on the amount of correlations: it shifts from $2S_{z}^{*}=-20$ for the case without correlations, to $2S_{z}^{*}=-16$ with two- and four-pair excitations included. Another characteristic point in this diagram is the minimum of energy of the domain-wall states, appearing for large $2S_{z}^{*}$. This minimum is related to the reconstruction of the edge of the droplet and is a finite size effect, which could not be eliminated by adjusting the single-particle energies in the Hamiltonian (A). This effect is not relevant for the understanding of the domain wall in the interior of the droplet.

The difference of the energy of the state with any $2S_{z}$ and the energy of the state $|UP\rangle$ (or $|SP\rangle$) can be interpreted as the energy needed to flip $S_{z}$ spins. This energy strongly depends on the position of the domain wall. In our case, however, we are interested in the energy to flip *one* electron spin, that is the difference of energies corresponding to $2S_{z}$ and $2S_{z}+2$. This energy also strongly depends on the position of the domain wall, and appears to be minimal close to the critical value of $2S_{z}^{*}=-16$ (corresponding to the energy maximum). This is the central result of this work: we have found a spin domain state, for which flipping one spin costs a small energy, possibly commensurate with the spin-flip energy of the nuclear spin, thus enabling the flip-flop process between the 2DEG and the nuclear spin. We will now focus on understanding the details of the electronic spin-flip process.

We start with analyzing the nature of the $2S_{z}^{*}=-16$ state. Figure 3(a) of the main text shows the expectation value of the spin projection as a function of the quantum number *m* of the relevant orbitals of the LLL and 2LL taking part in the construction of the domain: $\langle2S_{z}^{*}|\hat{S}_{z}\left(m\right)|2S_{z}^{*}\rangle=\langle2S_{z}^{*}|c_{0,m,\uparrow}^{+}c_{0,m,\uparrow}-c_{1,m,\downarrow}^{+}c_{1,m,\downarrow}|2S_{z}^{*}\rangle$. We show the single-configuration domain wall in black; in this case we see an abrupt change of spin between the spin-down domain (center of the droplet) and the spin-up domain (edge of the droplet). For this state $2S_{z}^{*}=-16$, the last orbital holding a spin-down electron is (*n,m*)=(1,7), and the spin-up domain begins with the orbital (*n,m*)=(0,8). As we include the two-pair (red line) and two- and four-pair excitations (blue line), the domain wall becomes softer, but it converges quickly with the number of pair excitations. We find therefore that the reversal of the spin between domains takes place over several single-particle orbitals. However, we are unable to visualize the rotation of the spin in space, as the state $2S_{z}^{*}=-16$ is the eigenstate of the $\hat{S}_{z}$ operator, and thus the expectation values of the operators $\hat{S}_{x}$ and $\hat{S}_{y}$ are zero.

We can now calculate the effective magnetic field experienced by the localized spin and generated by the electrons. To this end, we consider the expectation value of the last term of the Hamiltonian (A) as a function of the position $R$ of the nuclear spin (we place the spin on the x axis and move radially from the center of the droplet towards its edge). The effective Knight field is

$B_{z}^{KNIGHT}\left(R\right)=\langle2S_{z}^{*}=-16|\sum_{n=0}^{1} \sum_{m=0}^{N_{e}/2-1} \sigma\left|\phi_{nm}\left(R\right)\right|^{2}c_{n,m,\sigma}^{+}c_{n,m,\sigma}|2S_{z}^{*}=-16\rangle$.

Note that here we include the polarized background of electrons occupying the LLL with spin down. This local Knight field is plotted in Fig. 3(b) with a black line for the one-configuration $|2S_{z}^{*}=-16\rangle$ state, and with a red line for the state containing the two- and four-pair excitations. In this real-space representation we find that the effective Knight field is large close to the center of the droplet, and decreases to zero towards its edge, which is to be expected in our arrangement of the spin domains. Also, we find that the domain wall is in fact much broader than what might be expected from Fig. 3(a) owing to the spatial extent of the single-particle domains, which is not captured if we plot as a function of just the quantum number *m*.

**Domain wall states - variational calculations.**

The factorial growth of the Hilbert space size with the number of excitations makes it difficult to characterize the convergence of energies corresponding to the wavefunction $|S_{z},L_{z},k\rangle$ (Eq. 2 of the main text) as a function of the number of electron-hole pairs. Therefore, in order to test the quality of the four electron-hole-pair approximation we adopt the variational approach, utilized in studies of spin domain states in the quantum Hall filling factor 1 regime [22-26]. We look for the spin-domain state in the form $|\Psi_{0}\rangle=\prod_{m} \left[\cos\theta_{m} c_{1,m,\downarrow}^{+}+\sin\theta_{m}e^{i\phi_{m}} c_{0,m,\uparrow}^{+}\right]c_{0,m,\downarrow}^{+}|0\rangle$, in which the electron residing in each channel defined by the quantum number *m* is placed on an effective, variational orbital composed of the spin-orbitals belonging to the LLL and 2LL. This variational wavefunction has a broken spin and space symmetry, and can only be characterized by expectation values $\langle\Psi_{0}|\hat{S}_{z}|\Psi_{0}\rangle$ and $\langle\Psi_{0}|\hat{L}_{z}|\Psi_{0}\rangle$ of the total spin and angular momentum projections, respectively. In terms of excitations, the variational wavefunction may be perceived as a particular linear combination of all possible pair excitations, excluding doubly-occupied configurations. It also allows to define the local magnetization, $\hat{M}(m)=[(\sin2\theta_{m}\cos\phi_{m}, \sin2\theta_{m}\sin\phi_{m}, \cos2\theta_{m}]$, which describes the local electronic pseudospin as it rotates in space across the domain wall [24]. In our exact diagonalization approach we resolve the total spin projection $\hat{S}_{z}$ as a good quantum number, which leads to the expectation values of the two other spin components equal to zero across the system.

We find the variational wave function for each total spin polarization $S_{T}$ by minimizing the expectation value of the Hamiltonian (A), but without the electron-nuclear spin terms, and under the subsidiary condition $\langle\Psi_{0}|\hat{S}_{z}|\Psi_{0}\rangle=S_{T}$ . To this end, we look self-consistently for the minimum of the free energy , with the Lagrange parameter $\lambda$ playing a role of the “Zeeman potential”. This is equivalent to the self-consistent Hartree-Fock procedure, in which additionally we adjust the value of $\lambda$ to maintain the desired total spin projection in each self-consistent step.

The variational energies of our system as a function of $S_{T}$ are shown in Figure 2(b) of the main text with the green crosses. We find a good agreement of these energies with the results of the exact diagonalization with up to four electron-hole pair excitations. In particular, we find the maximum energy as a function of the spin projection at $2S_{z}=-16$, just as it is in the exact diagonalization result. Further, close to this energy maximum the energies obtained in the exact diagonalization (blue lines) are even slightly lower than those from the variational approach, while at large values of the spin projection (the right-hand side of the graph) the variational energies appear to be somewhat better than the four-pair results.

We have also analyzed the local magnetization $\vec{M}\left(m\right)$ for the variational state with $2S_{T}=-16$ and found that the component $M_{z}\left(m\right)$ of that vector traces the domain wall very similar to that shown in Fig. 3(a) of the main text for the four-pair approximation (blue). We therefore conclude that the wavefunction $|S_{z},L_{z},k\rangle$ containing up to four electron-hole pair excitations captures the essential physics of the spin domain states satisfactorily.

**Electronic spin flip.**

As already mentioned, our state $|2S_{z}^{*}=-16\rangle$ is an eigenstate of the total spin projection operator $\hat{S}_{z}$. The spin flip-flop process between the electronic and the nuclear spins must result in a change of the state of our electrons, i.e., spin flip to the state $|2S_{z}=-14\rangle$ or $|2S_{z}=-18\rangle$. In what follows we shall focus on the former case, which is summarized in Fig. 4 of the main text. We switch from the state $|2S_{z}^{*}=-16\rangle$, with the largest energy in Fig. 4(a), and depicted schematically in the right-hand diagram of Fig. 4(b), to the state $|2S_{z}=-14\rangle$, expecting one spin flip at the domain wall (Fig. 4(a)). The final state, depicted schematically in the left-hand diagram of Fig. 4(b), is also a domain-wall state, but with the domain wall shifted by one orbital towards the center of the droplet. In this transition, the energy of the electronic system will decrease by an amount which can be made commensurate to the Zeeman splitting of the nuclear spin. As a result, the nuclear spin, residing at position $R$, is expected to take up the excess energy and flip its spin by one unit down, as depicted schematically in Fig. 4(b). The probability of this flip-flop process is calculated as the matrix element of the first term of the electron-nuclear interaction part of the Hamiltonian (1):

.

Here we choose to place the nuclear spin at the position $R$ such that it resides at the peak of the subsequent LLL single-particle orbitals, and we characterize this position by an integer number $m_{R}=R^{2}/2l_{h}^{2}$ which is the quantum number *m* of that LLL orbital. Figure 5 shows the reduced amplitude $4I\left(m_{R}\right)/(J_{0}^{2}|\phi_{0m*}(R)|^{2}\left[M\left(M+1\right)-M_{Z}\left(M_{z}-1\right)\right])$ as a function of the position $m_{R}$ of the nuclear spin. We note that this amplitude is exactly zero at the orbital corresponding to the center of the domain wall ($m_{R}=7$). This results from the form of the single-particle orbitals. Indeed, if we consider the spin-flip transition between the fundamental spin-domain configurations, as presented in Fig. 4(b), the above amplitude would be proportional to the term $\left|\phi_{0,7}^{*}\left(R\right)\phi_{1,7}\left(R\right)\right|^{2}$. The functional form of the orbitals, assuming that the nuclear spin is positioned along the *x* axis, is respectively $\phi_{0,7}\left(R\right)=\frac{1}{l_{h}\sqrt{2\pi7!}}\left(\frac{R}{\sqrt{2}l_{h}}\right)^{7}e^{-R^{2}/4l_{h}^{2}}$ and $\phi_{1,7}\left(R\right)=\frac{1}{l_{h}\sqrt{2\pi7!}}\left(\frac{R}{\sqrt{2}l_{h}}\right)^{6}\left(7-\frac{R^{2}}{2l_{h}^{2}}\right)e^{-R^{2}/4l_{h}^{2}}$. As we can see, as the LLL orbital has a maximum for $R=l_{h}\sqrt{2m_{R}}$ with $m_{R}=7$, the 2LL orbital has a node for this coordinate. Thus, the spin flip cannot be carried out when the nuclear spin is placed in the center of the orbital of the electron undergoing the transition. However, this cancellation manifestly does not occur for any other position of the nuclear spin, and the transition amplitude increases with the coordinate $R$ either to the left or to the right of the domain wall, to reach its maximum and then decay exponentially as the nuclear spin is moved deeper within the spin domains.

In Fig. 5 of the main text, with black line we present the amplitude calculated for the states composed of the single spin-domain configurations only, i.e., we trace the simple product of the single-particle orbitals given above. This amplitude is normalized to the value $\left|\phi_{0,7}^{*}\left(R\right)\phi_{0,7}\left(R\right)\right|^{2}$, i.e., the orbital factor expected if the spin were to be flipped without changing the Landau level. As we add the correlations (blue line), we see that the amplitude is also zero when the nuclear spin is placed at the center of the domain wall, but the amplitude is enhanced for all other positions of the nuclear spin.
